# Epilepsy: neuroinflammation, neurodegeneration, and *APOE* genotype

**DOI:** 10.1186/2051-5960-1-41

**Published:** 2013-07-29

**Authors:** Orwa Aboud, Robert E Mrak, Frederick A Boop, W Sue T Griffin

**Affiliations:** 1 Donald W. Reynolds Department of Geriatrics, University of Arkansas College of Medicine, Little Rock, AR 72205, USA; 2 Department of Neurobiology and Developmental Sciences, University of Arkansas College of Medicine, Little Rock, AR 72205, USA; 3 Department of Pathology, University of Toledo Health Sciences Campus, Toledo, OH 43614, USA; 4 University of Tennessee Health Sciences Center, Memphis, TN 38103, USA; 5 The Geriatric Research Education Clinical Center Central Arkansas Veterans Healthcare Systems, Little Rock, AR 72205, USA; 6 Department of Geriatrics, Donald W. Reynolds Institute on Aging, #4103, 629 Jack Stephens Drive, Little Rock, AR 72205, USA

**Keywords:** Alzheimer’s disease, Apolipoprotein E (ApoE), *APOE* genotype, Caspase 3, Epilepsy, Neuroinflammation

## Abstract

**Background:**

Precocious development of Alzheimer-type neuropathological changes in epilepsy patients, especially in *APOE* ϵ4,4 carriers is well known, but not the ways in which other *APOE* allelic combinations influence this outcome. Frozen and paraffin-embedded tissue samples resected from superior temporal lobes of 92 patients undergoing temporal lobectomies as a treatment for medication-resistant temporal lobe epilepsy were used in this study. To determine if epilepsy-related changes reflect those in another neurological condition, analogous tissue samples harvested from 10 autopsy-verified Alzheimer brains, and from 10 neurologically and neuropathologically normal control patients were analyzed using immunofluorescence histochemistry, western immunoblot, and real-time PCR to determine genotype effects on neuronal number and size, neuronal and glial expressions of amyloid β (Aβ) precursor protein (βAPP), Aβ, apolipoprotein E (ApoE), S100B, interleukin-1α and β, and α and β secretases; and on markers of neuronal stress, including DNA/RNA damage and caspase 3 expression.

**Results:**

Allelic combinations of *APOE* influenced each epilepsy-related neuronal and glial response measured as well as neuropathological change. *APOE* ϵ3,3 conferred greatest neuronal resilience denoted as greatest production of the acute phase proteins and low neuronal stress as assessed by DNA/RNA damage and caspase-3 expression. Among patients having an *APOE* ϵ2 allele, none had Aβ plaques; their neuronal sizes, like those with *APOE* ϵ3,3 genotype were larger than those with other genotypes. *APOE* ϵ4,4 conferred the weakest neuronal resilience in epilepsy as well as in Alzheimer patients, but there were no *APOE* genotype-dependent differences in these parameters in neurologically normal patients.

**Conclusions:**

Our findings provide evidence that the strength of the neuronal stress response is more related to patient *APOE* genotype than to either the etiology of the stress or to the age of the patient, suggesting that *APOE* genotyping may be a useful tool in treatment decisions.

## Background

Epilepsy is the third most common cause of neurological disability worldwide [1] and is associated with precocious development of the neuropathological changes of Alzheimer’s disease (AD) [2-4]. Traumatic brain injury (TBI), which is a major risk factor for the development of epilepsy [5], is also associated with increased risk for later development of AD, and, in both cases, the risk of development of AD is greater with inheritance of apolipoprotein E ϵ4 alleles (*APOE* ϵ4) [3,6]. Exploring possible links between epilepsy-related alterations in neuronal and glial cell responses relative to *APOE* genotype is potentially important in understanding chronic neurodegenerative sequelae in epilepsy as well as in other forms of brain injury such as TBI [5,7-10].

In both purified rodent neuronal cell cultures and cultures of the human neuroblastoma cell line NT2, excess glutamate induces marked increases in the expression of the neuronal acute phase response protein βAPP, and in the release of its secreted fragment sAPPα [11], which is a powerful inducer of glial activation and increased production and release of the proinflammatory cytokine IL-1β [12]. This sAPP-induced glial activation and cytokine expression and release is differentially modulated in the presence of ApoE3 vs ApoE4 [11], with ApoE3 providing greater protection than ApoE4. In epilepsy, there is overexpression of βAPP and IL-1, as well as the astrocyte-derived, neuritogenic cytokine S100B [13,14]. Furthermore, a comparison between surgical waste tissues from patients undergoing anterior temporal lobectomy surgery for drug-resistant intractable epilepsy showed that *APOE* ϵ3,3 and *APOE* ϵ4,4 genotypes dramatically alter the expression of βAPP and of IL-1 such that the *APOE* ϵ3 allele is more effective with regard to the maintenance of appropriate neuronal acute phase responses that favor neuronal viability than is *APOE* ϵ4 [2]. Importantly, several studies have provided evidence that inheritance of an *APOE* ϵ4 allele is associated with increased risk for Alzheimer neuropathological changes in epilepsy patients [3]. This is particularly relevant to the possibility that the decrease in the ability of ApoE4 compared with ApoE3 to elevate synthesis of the neuronal acute phase protein βAPP [15] is responsible for less sAPPα release, resulting in diminished neuronal repair and survival [16].

Epilepsy, in particular, exemplifies the intimate relationship between neuronal stress and triggering of glial activation, as such interactions are self-amplifying in epilepsy. For example, glutamate-induced hyperexcitation in purified primary rat neurons results in increases in βAPP, sAPP, and IL-1β, as well as ApoE, and both IL-1β and ApoE induce βAPP expression and sAPP release. Moreover, IL-1β treatment of neurons results in glutamate release [17], promoting a proposed self-perpetuating series of events: glutamate → ApoE → βAPP → sAPP → IL-1β → glutamate. Initially, cycle-engendered early acute phase responses may be beneficial, affording neuronal protection and debris clearance. However, because of the self-perpetuating nature of this cycle and the resultant glutamate release from both glia [18] and neurons [17] chronic neuronal stress ensues, enhancing the probability of neurodegeneration. Furthermore, the potential of such a cycle to be self-regenerative may explain, at least in part, why even those epilepsy patients with the advantage of an *APOE* ϵ3,3 genotype may develop Alzheimer-type neuropathological changes.

Evidence of a role for *APOE* genotype in determining neurodegenerative consequences of epilepsy underscores the need for in-depth analyses of neuronal-glial interactions that may be governed by inheritance of specific *APOE* allelic combinations. Such analyses provide basic cellular and molecular information regarding pathways involved in neuronal-glial interactions as well as information that may be helpful in clinical decision making.

## Results

Comparison between our patient groups – Group 1: patients having one or two alleles of ϵ2 and no ϵ4 [*APOE* ϵ2,2 (n = 1) and *APOE* ϵ2,3 (n = 12)] age range = 14 y-73 y, median age = 40 y, average age = 35 y; Group 2: [*APOE* ϵ3,3 patients (n = 53)], age range = 0.25 y-71 y, median age = 32 y, average age = 30.4 y; Group 3: patients having one ϵ4 allele [*APOE* ϵ2,4 (n = 2) *APOE* ϵ3,4 (n = 17)], age range = 10 y-48 y, median age = 30.5 y, average = 29.2 y; Group 4: [*APOE* ϵ4,4 patients (n = 7)], age range = 10 y-50 y, median age = 34 y, average age = 32 y. Such grouping allowed for investigation of the degree of influence of each of the 6 combinations of the three *APOE* alleles and provided the following results.

### *APOE* genotype modulation of glial responses in epilepsy

#### Glial numbers in a given cross-sectional cortical area

**Figure 1:**
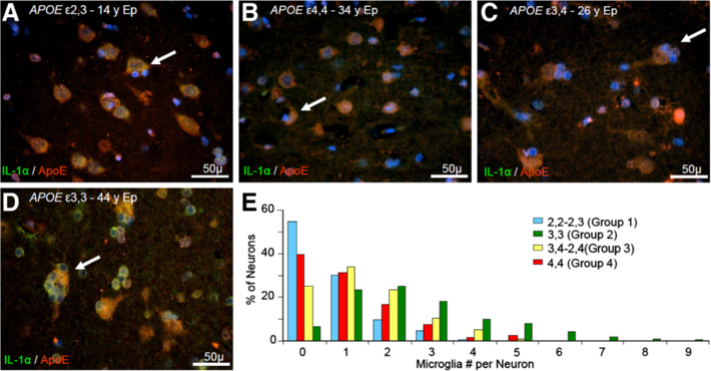
**The relationship between neuronal expression of ApoE and glial expression of IL-1α, relative to *APOE *genotype.** IL-1α (green)-immunoreactive microglia clustered around individual ApoE-immunoreactive neurons (red) in patients with *APOE* ϵ2,3 **(A)**, *APOE* ϵ4,4 **(B)**, *APOE* ϵ3,4 **(C)**, and *APOE* ϵ3,3 **(D)**. A maximum of 9 microglia per neuron were counted in *APOE* ϵ3,3 patients (arrow in **D**); this was higher than the numbers associated with other *APOE* genotypes as shown in the percentage of neurons with adjacent IL-1α immunoreactive neurons (none to nine) relative to *APOE* genotype **(E)**.

Patients in Group 1 (patients having one or two alleles of ϵ2 but no ϵ4) had the lowest number of microglia per neuron: 82% of the neurons had less than two adjacent microglia. Relative to other groups, Group 2 (patients with two *APOE ϵ*3 alleles ϵ3,3) had more IL-1α-immunoreactive microglia adjacent to each neuron: 70% of the neurons had at least two adjacent microglia, with some having as many as 9. In Group 3 (patients having one *APOE* ϵ4 allele, ϵ2,4 and ϵ3,4), more than 80% of neurons had two or less IL-1α immunoreactive microglia per neuron. In Group 4 (patients having two *APOE* ϵ4 alleles, ϵ4,4), 70% of neurons had one or fewer adjacent microglia (Figure 1).

In a given unit area of cortical layers III and IV of superior temporal gyrus in patients in Group 2 (patients having two *APOE* ϵ3 alleles), the total number of microglia counted was greater than that in other groups (Group 2 = 1137 ± 267 vs Group 1 = 460 ± 112; Group 3 = 534 ± 81; Group 4 = 770 ± 187 microglia/mm^2^; p < 0.001). Although the number of microglia/mm^2^ was influenced by *APOE* genotype, the relative levels of glial cytokine mRNAs IL-1α and IL-1β were not related to genotype (data not shown).

**Figure 2:**
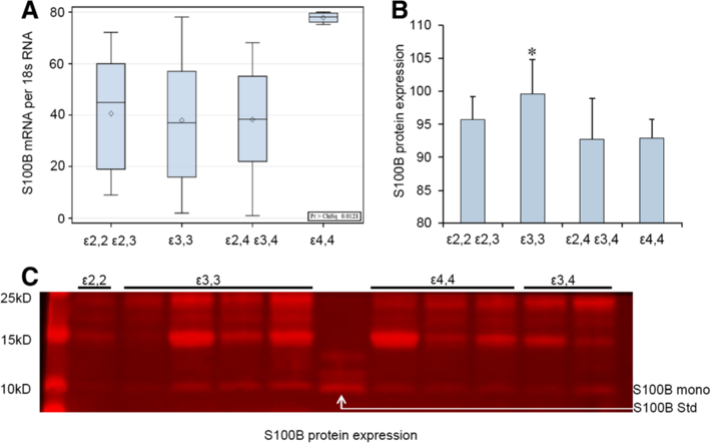
***APOE *genotype influences expression of the astrocyte-derived neuritogenic cytokine S100B.** Wilcoxon distribution scores for S100B mRNA levels in patients with *APOE* ϵ4,4 (Group 4) is higher than that in other genotypes. p values: Group 4 vs 1, p = 0.004: Group 4 vs 2, p = 0.001; and Group 4 vs 3, p = 0.004 **(A)**. S100B protein levels for all the patient samples (n = 92), quantified by western blot, showed that *APOE* ϵ3,3 patients (Group 2, n = 53) had higher levels of S100B than did other *APOE* genotypes (99.7 ± 5.17 vs 95.7 ± 3.55, 92.7 ± 6.21, 92.9 ± 2.87 arbitrary units; p = 0.001) for groups 1 (n = 13), 3 (n = 19), and 4 (n = 7), respectively **(B)**. Illustration of S100B protein levels; one of eight western blots of different epilepsy samples (n = 92) with standard (Std) purified S100B positive control (middle sample S100B Std) the S100B mono band represents the S100B monomer (~11kD) **(C)**.

Astrocyte numbers per unit area were highest in patients with *APOE* ϵ3,3 (Group 2) compared to those in patients with other *APOE* allelic combinations, groups 1, 3, and 4 respectively (175 ± 19 vs 94 ± 38; 96 ± 12; 133 ± 12 astrocyte/mm^2^; p < 0.001). However, synthesis of the astrocyte-derived cytokine S100B was higher in patients with *APOE* ϵ4,4 compared to that in patients carrying other allelic combinations (Figure 2A). Conversely, S100B protein expression was higher in *APOE* ϵ3,3 (Group 2) patients (99.7 ± 5.17) than in groups 1, 3, or 4, respectively (95.7 ± 3.55, 92.7 ± 6.21, 92.9 ± 2.87; p < 0.001) (Figure 2B and C).

### *APOE* genotype modulation of neuronal responses in epilepsy compared to that in Alzheimer patients

#### Neuronal numbers in a given cortical area and neuronal cross-sectional area in epilepsy

**Figure 3:**
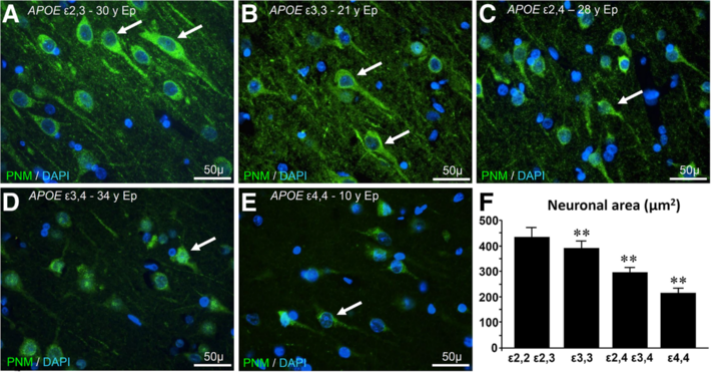
**Epilepsy-induced neuronal stress affects neuronal size in an *APOE *genotype-dependent fashion.** Neurons in tissue from epilepsy patients were immunoreacted with a pan neuronal marker (green). The apparent large size of neurons in patients with *APOE* ϵ2,3 **(A)** and those in patients with *APOE* ϵ3,3 **(B)** relative to those in patients with *APOE* ϵ2,4 **(C)**, *APOE* ϵ3,4 **(D)**, and *APOE* ϵ4,4 genotype **(E)** was confirmed by measuring cross-sectional areas of neurons (μm^2^) in Groups 1,2,3,4, respectively (432 ± 40; 389 ± 29; 294 ± 20; vs 213 ± 17; p < 0.001) **(F)**.

As previously reported [2], we observed no differences in neuronal numbers between epilepsy patients with either an *APOE ϵ*3,3 or *APOE ϵ*4,4 genotype. However, the cross-sectional area of neurons from patients in Group 1 as well as Group 2 (those having one or two *APOE* ϵ2 alleles, but no *APOE* ϵ4 allele, or those having *APOE* ϵ3, 3 genotype, respectively) was greater (439 ± 32 μm^2^ vs 389 ± 29 μm^2^). *APOE ϵ*4,4 neurons (Group 4) had the smallest neuronal area (213 ± 17 μm^2^) among the Groups, p < 0.001 (Figure 3).

#### Neuronal numbers in a given cortical area and neuronal cross-sectional area in Alzheimer patients vs control patients

**Figure 4:**
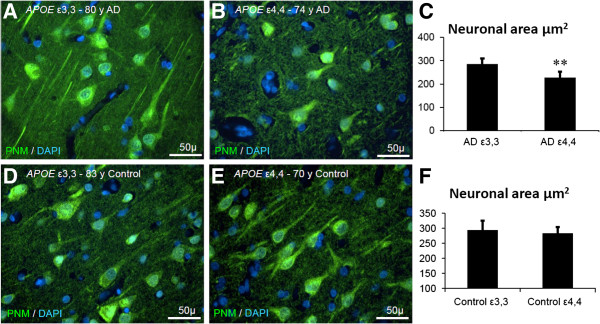
**Cross sectional neuronal area in Alzheimer patients and in neurologically normal controls, relative to *APOE *genotype.** Superior temporal gyrus tissue sections from Alzheimer patients **(A-C)** and neurologically normal patients **(D-F)** immunoreacted with Pan neuronal marker (green) in Alzheimer patients having *APOE* ϵ3,3 **(A)** appeared to be larger than those from Alzheimer patients with *APOE* ϵ4,4 **(B)**. Cross-sectional area measurements of neurons (μm^2^) confirm this difference (286 ± 23, vs 227 ± 25; p = 0.009) **(C)**. Cross-sectional area of neurons in control patients with *APOE* ϵ3,3 **(D)** was not different from that in control patients with *APOE* ϵ4,4 **(E)** (250 ± 16, vs 244 ± 28; p = 0.67) **(F)**.

In tissue from patients with Alzheimer’s disease, the sizes of *APOE* ϵ3,3 neurons were larger than those of *APOE ϵ*4,4 neurons (286 ± 23 μm^2^ vs 227 ± 25 μm^2^, p < 0.01) (Figure 4A-C). In contrast, the size of neurons in analogous tissue from neurologically normal controls did not differ with A*POE* genotype (*APOE* ϵ3,3 neurons, 293 ± 32 μm^2^ and *APOE ϵ*4,4 neurons, 282 ± 21 μm^2^) (Figure 4D-F). The cross-sectional area of *APOE* ϵ4,4 neurons was less than that of *APOE* ϵ3,3 neurons in both epilepsy and Alzheimer patients, perhaps suggesting that *APOE* ϵ4,4 neurons are not able to follow the same response to neuronal stress as do neurons of other genotypes. No *APOE* genotype-related changes in neuronal cell numbers were detected in our epilepsy patient Groups 1,2,3, and 4 (302 ± 39, 277 ± 25, 280 ± 32, 272 ± 21 per mm^2^, respectively).

#### Neuronal impairment in epilepsy

**Figure 5:**
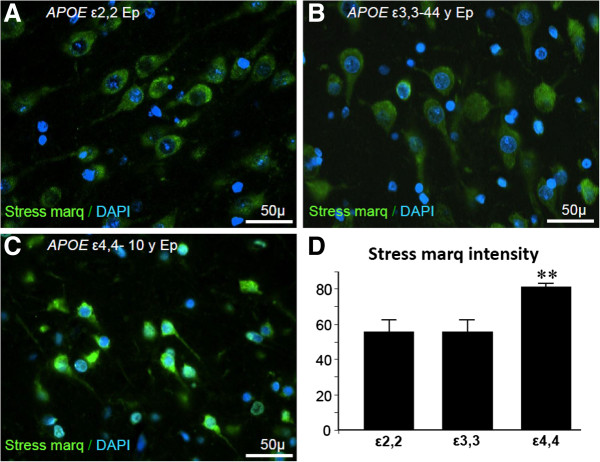
**Neuronal stress as estimated by heat shock-related damage associated with expression of 8-OH guanosine.** Numbers of neurons expressing RNA/DNA oxidative damage was similar in the different APOE genotype groups **(A-C)**. However, the extent of damage per neuron, as assessed by Stress Marq (8-OH guanosine) fluorescence intensity, was greater in patients with *APOE* ϵ4,4 than *APOE* ϵ2,2 or ϵ3,3 genotypes (80 ± 3 vs 55 ± 6 and 55 ± 6 arbitrary units, p = 0.001) **(D)**.

**Figure 6:**
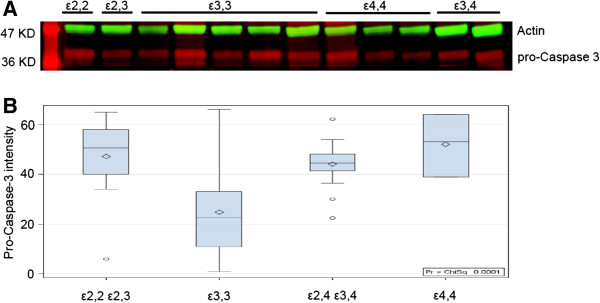
***APOE *genotype influences cell cycle-related DNA damage.** Illustration of a western blot representative of 8 gels from patients (n = 92) with different APOE genotypes, showing actin bands (green) and pro-caspase 3 bands (red) **(A)**. Wilcoxon distribution scores for pro-caspase 3 fluorescence intensity for all the 92 patients: *APOE* ϵ3,3 (Group 2) had lower levels of pro-caspase than other genotypes **(B)** with p values for comparisons with Groups 1,3,4 equal to < 0.002, < 0.001, and < 0.02, respectively.

*APOE* genotype-related RNA/DNA oxidative damage, measured as 8-OH guanosine intensity was examined in a small sample of our patients that was somewhat reflective of the rarity of *APOE* genotypes among our patients and among the general population [*APOE ϵ2*,2 (n = 1); *APOE ϵ*3,3 (n = 10); and *APOE ϵ*4,4 (n = 4) genotypes]. This analysis showed that there was less oxidative damage to DNA in tissue from epilepsy patients with genotypes other than *APOE ϵ*4,4 (55 ± 6 and 55 ± 6 vs 80 ± 3 arbitrary units, respectively, p < 0.01) (Figure 5), even though this indication of cellular stress is not necessarily followed by DNA fragmentation [19]. Assessment of epilepsy-related neuronal cell death pathways (measured by western blot analysis of caspase 3 levels in all samples) showed that caspase 3 levels were lower in *APOE ϵ*3,3 patients (Group 2) than in Groups 1, 3, or 4, respectively (8.4 ± 4.1 vs 13.2 ± 4.0, 11.7 ± 2.4, 14.4 ± 4.0, p < 0.05) (Figure 6).

#### Acute phase responses in epilepsy relative to APOE genotype

**Figure 7:**
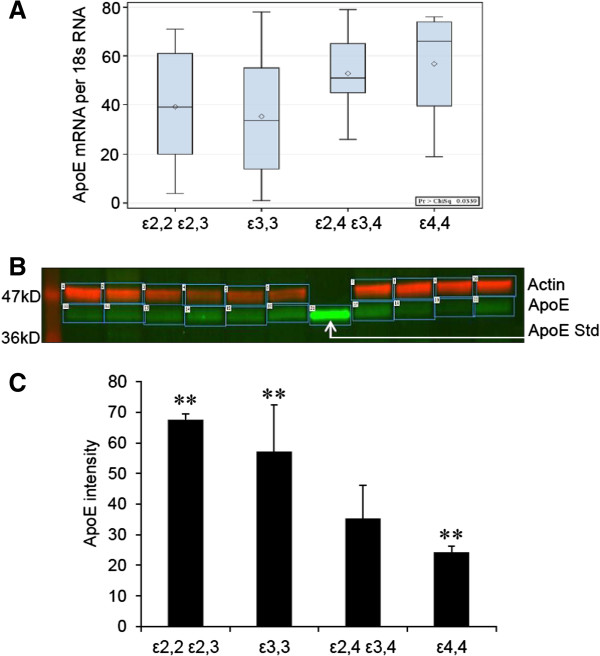
**ApoE transcription and translation relative to *APOE *genotype.** Wilcoxon distribution scores for ApoE mRNA levels were higher in patients carrying at least one *APOE* ϵ4 allele (Group 3 and Group 4) than in those carrying other APOE genotypes (Groups 1 and 2); the difference was significant only between Group 2 and Group 3 (p < 0.01) **(A)**. ApoE protein levels, illustrated in one of eight western blots of different epilepsy samples (n = 92) with actin (red) in the upper row and ApoE (green) in the lower row with the middle sample being recombinant ApoE protein used as a positive control (Std) **(B)**. ApoE protein levels for all the patient samples (n = 92), quantified by western blot analysis, showed that *APOE* ϵ4,4 patients (Group 4) had lower levels of ApoE than did other *APOE* genotypes (18.2 ± 8.4 vs 61.8 ± 15.3, 55.4 ± 23.3, 26.9 ± 7.1 arbitrary units) for groups 1, 2, and 3, respectively, p < 0.01 **(C)**.

To assess the neuronal response to the hyperexcitation stress in epilepsy, we measured the relative tissue levels of the messenger RNAs for both βAPP and ApoE in tissue samples from patients (n = 92) with each of the different *APOE* allelic combinations, using real time PCR analysis. βAPP mRNA expression did not show significant differences with regard to *APOE* genotype (data not shown). However, ApoE mRNA levels were higher in Group 4 (the *APOE* ϵ4,4 group) than in other groups (Figure 7A). Despite the lack of increased expression of βAPP mRNA among our patients, there was a dramatic increase in the levels of βAPP protein in those with *APOE* ϵ3,3 (Group 2) compared to those levels in *APOE* ϵ4,4 (Group 4), suggesting that the elevation of βAPP in this group was due more to translation than transcription. Conversely, ApoE protein levels, measured by western blot analysis showed that *APOE* ϵ4,4 patients (Group 4) had lower levels than did other *APOE* genotypes (18.2 ± 8.4 vs 61.8 ± 15.3, 55.4 ± 23.3, 26.9 ± 7.1; Group 4 vs 1, 2, and 3, respectively, p < 0.01) (Figure 7B,C), suggesting that even the increase in ApoE mRNA in tissues samples from Group 4 patients was not sufficient to raise ApoE protein levels to those noted in other groups. Moreover, Group 4 (*APOE* ϵ4,4) patients had lower tissue levels of actin than did patients in Groups 1, 2, and 3, respectively (57.0 ± 13.0 vs 83.8 ± 13.0, 114.3 ± 39.5, 106.1 ± 19.0, p < 0.01). This may explain, at least in part, the differences noted in neuronal size. For example, without regard to cell type, size may influence actin levels. Alternatively, actin levels may influence cell size, as actin has been used to estimate cell size in some studies [20].

### The influence of *APOE* genotypes on epilepsy-induced neuropathological changes

**Figure 8:**
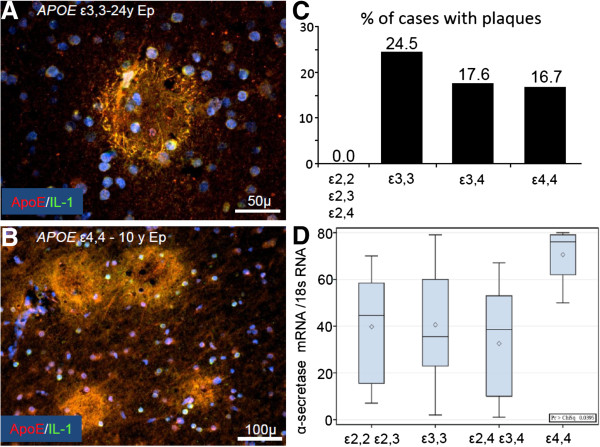
**Neuropathological changes in epilepsy relative to *APOE *genotype.** Multiple IL-1α (green)/ApoE (red)-immunoreactive plaques were present in 13 of the 53 *APOE* ϵ3,3 patients **(A)** – the youngest of which was 17 yrs old – and in 3 of the 17 *APOE* ϵ3,4 patients (not shown here). One of the six *APOE ϵ4,4* patients had Aβ plaques, a 10 year old male **(B)**. The incidence of plaques in patients having each of the *APOE* allelic subtypes **(C)**. Wilcoxon distribution scores for α-secretase mRNA measured relative to 18s mRNA showed that *APOE* ϵ4,4 patients (Group 4) had higher levels than did other *APOE* genotypes, with p values of 0.02, 0.01, and 0.01 for comparisons between group 4 and groups 1, 2, and 3, respectively **(D)**.

None of the 15 patients having at least one *APOE* ϵ2 allele had Aβ plaques. This is in contrast to the fact that Aβ plaques were present in carriers of all other genotypes [13 of the 53 patients with *APOE* ϵ3,3, 24.5%; 3 of 17 patients with *APOE* ϵ3,4, 17.6%; and in 1 of the 6 patients with *APOE* ϵ4,4, 16.7% (one of the seven *APOE* ϵ4,4 patients was not examined immunohistochemically because of a limited amount of tissue)] (Figure 8A-C). These findings raise the possibility of a protective role of *APOE* ϵ2 alleles against plaque formation in epilepsy. The elevated plaque density in those with *APOE* ϵ3,3 and *APOE* ϵ3,4 compared to other genotypes is consistent with the findings in these patients of elevated ApoE and S100B; both induce elevation of βAPP expression, which is then available for Aβ cleavage and deposition. Although *APOE* genotype did not influence expression of the mRNAs for the β-secretases (BACE1 and 2), α-secretase mRNA expression was elevated in those with *APOE* ϵ4,4 genotype (Group 4) compared to other genotypes (Figure 8D). The *APOE* ϵ4,4-related increase in α-secretase mRNA expression might be viewed as an attempt at compensation for other deficiencies as α-secretase obviates the production of Aβ and at the same time increases secretion of sAPPα, which may provide a neuron-sparing action [16]. It is interesting to note that in epilepsy with the early appearance of Aβ plaques and glial activation, there is little or no evidence of neurofibrillary tangle formation [21].

## Discussion

Our results show that, compared to other *APOE* allelic combinations, neuronal resilience and glial activation were greatest in epilepsy patients with *APOE* ϵ3,3 genotype. Neuronal resilience correlated with the highest expression of acute phase response proteins βAPP and ApoE, greatest neuronal size, and least indications of DNA fragmentation, oxidation, and potential cell cycling. Similarly, compared to patients with other allelic combinations, patients with *APOE* ϵ3,3 genotype had more IL-1α-immunoreactive microglia in a given cross-sectional cortical area, more microglia adjacent to neurons, and more astrocytes per given cross-sectional area. In addition, the tissue levels of the astrocyte-derived, neuron-sparing neuritogenic cytokine S100B were highest in patients with *APOE* ϵ3,3 genotype. These findings are consistent with the idea that *APOE* genotype influences neural responses to the neuronal stress engendered by the hyperexcitation of epilepsy. Several neural stresses elicit elevation of neuronal acute phase proteins βAPP and ApoE, which, in turn, promote microglial and astrocytic activation with increased expression of glial cytokines such as IL-1β and S100B. These cytokines are known to regulate the expression of each other and both are known to induce the expression of βAPP and ApoE for promotion of neuronal survival and maintenance [15,17,22-24]. Together, our findings are consistent with the idea that hyperexcitation first elicits compensatory responses that include overexpression of βAPP, release of sAPP, and glial activation with induction of IL-1 and S100B. The fact that carriers of one or two *APOE* ϵ3 allele(s) were shown here to be more adept than carriers of other *APOE* genotypes at eliciting specific neuronal and glial responses that have been associated with neuronal repair and survival [16,25] suggests that neuronal resilience is, at least in part, dependent on which specific ApoE variant is present.

Our findings that inheritance of two *APOE* ϵ4 alleles is associated with smaller neurons in both epilepsy and Alzheimer’s disease, but not in neurologically normal controls, together with the dramatic elevation of markers of stress in *APOE* ϵ4,4 carriers, suggests that such individuals are at greater risk of neuronal damage, regardless of the initiating injury. Elevated expression of ApoE mRNA, in conjunction with a lower expression of ApoE protein in those with *APOE* ϵ4,4 genotype may represent a failed attempt to increase ApoE expression as a way of increasing βAPP expression, which in itself may be futile as ApoE4 has been shown to be an ineffective stimulant of βAPP expression [15].

The Aβ plaques and hippocampal atrophy in temporal lobectomy tissues from epilepsy patients have been observed in other studies [4,26]. Our finding that the incidence of Aβ plaques is dependent on specific *APOE* allelic subtypes, namely, that having even one *APOE* ϵ2 allele is associated with an absence of Aβ plaques even in our oldest patient (71y), may in some way be related to a reported decreased risk for AD [27] and a protective effect of inheritance of *APOE* ϵ2 against Alzheimer-like neuropathological changes [28]. The Aβ plaques, which were noted in our 10-year old *APOE* ϵ4,4 patient, appeared to be mature dense core neuritic plaques, while those in carriers of other allelic combinations appeared as diffuse neuritic plaques. These findings support previous studies showing a relationship between the presence of an *APOE* ϵ4 allele and precocious development of AD in epilepsy patients [3,29], and suggests the need for further investigation into the role of *APOE* ϵ4 alleles in Aβ plaque maturation. These findings may have relevance to the fact that plaque maturity is associated with both formation of Aβ dense cores as well as increases in ApoE immunoreactivity as Aβ plaques mature [30].

The fact that most of the Aβ plaques present in our patients were found in APOE ϵ3,3 carriers may, in principle, be mostly related to increases in βAPP expression, especially in view of concomitant increases in proteins, cytokines, and neurotransmitters that are known to induce increases in neuronal βAPP, viz., ApoE [15], IL-1 [17], S100B [23], and glutamate [17], perhaps identifying mechanisms by which the advantage of having APOE ϵ3 alleles may be accompanied by the disadvantage of fostering Aβ deposition. Interestingly, with regard to *APOE* genotype and Alzheimer neuropathological change, carrying even one *APOE ϵ*2 allele appears to have a protective effect against the formation of Aβ plaques, regardless of age and sex. These interpretations of our results are predicated on the idea that Aβ plaques are the consequence of hyperexcitation-induced, neuronal stress-related cycles that are important in the neuropathological progression observed in epilepsy and which occur as a consequence of disease severity and duration, perhaps regardless of genotype, age, and sex. Our observation regarding greater plaque maturity among those with *APOE* ϵ4,4 relative to other *APOE* genotypes is consistent with observations in Alzheimer patients [31,32] and in mice [33], which show that ApoE binding to Aβ disrupts Aβ clearance across the blood-brain barrier in an isoform-specific manner, with ApoE4 having a greater disruptive effect than ApoE3 or ApoE2 [33].

The lower tissue levels of actin in patient carriers of *APOE* ϵ4,4 compared to those with other *APOE* allelic combinations may explain, at least in part, the *APOE* ϵ4,4-related smaller neuronal cell size [20]. Variation in actin expression according to *APOE* genotype, as we show here, suggests that actin should not be used as a tool for normalizing the relative expression of proteins in situations in which actin varies with specific parameters.

## Conclusions

Our finding that even one *APOE* ϵ2 allele is protective against Aβ plaque deposition is consistent with previous reports associating this genotype with protection against Alzheimer’s disease [34]. The robust neuronal-glial response to the neuronal stress of epilepsy in *APOE* ϵ3,3 carriers suggests that overall they have an advantage over other genotypes as indicated by an ability to increase neuronal acute phase protein expression, a greater neuronal size, and increased resilience, as indicated by lower levels of markers for RNA and DNA damage, and lower susceptibility to inappropriate cell cycling and death pathways. However, the fact that the percentage of patients with Aβ plaques was highest in *APOE* ϵ3,3 carriers suggests that these beneficial effects occur at the expense of an increase in the possibility of cleavage of excess βAPP and formation of Aβ, as well as increasing deposition of ApoE in such plaques and in this way perhaps favoring plaque formation. *APOE* ϵ4,4 carriers in our study were disadvantaged compared to other genotypes, having the smallest neurons among the genotypes, lowest acute phase responses, and highest markers of stress. Taken together, our findings suggest that *APOE* genotype may be important in decisions regarding timing of surgical intervention for intractable epilepsy, as well as in decisions regarding exposure of individuals to activities with high risk for TBI.

## Methods

### Patients and specimens

#### Epilepsy tissue samples

Resected temporal lobe tissues were obtained from 92 epilepsy patients (58 males and 34 females; 1 *APOE ϵ2,2,* 12 *APOE ϵ2,3*, 53 *APOE ϵ3,3*, 2 *APOE ϵ2,4*, 17 *APOE ϵ3,4* and 7 *APOE ϵ4,4*) with an age at surgery ranging from 0.25 to 73 years (median age = 32 y, average age = 31.7 y). All patients underwent anterior temporal lobectomy for treatment of medication-resistant intractable epilepsy. For more information on patients regarding neuropathological evaluation please see [35]. Surgical waste obtained from the anterior portion of the superior temporal gyrus, an area some distance from sclerotic areas and epileptogenic foci, was dissected at 4 mm intervals, and alternate sections were preserved by flash freezing for molecular analyses and by formalin fixation for histological evaluation. For uniformity, immunohistochemical examination was restricted to cortical layers III and IV of superior temporal gyrus.

#### Autopsy tissue samples

Temporal lobe tissues analogous to that collected from epilepsy surgical waste was collected from autopsied brain tissue of neuropathologically diagnosed Alzheimer patients [n = 6 males and n = 4 females; 5 *APOE* ϵ3,3 (M-60 y; M-73 y; F-76 y; F-80 y; F-88 y) and 5 *APOE* ϵ4,4 (F-72 y; M-74 y; M-74 y; M-82 y; M-86 y]. Analogous tissue samples were also obtained from neurologically and neuropathologically normal individuals [n = 8 males and n = 2 females; 6 *APOE* ϵ3,3 (M-69 y; M-78 y; M-78 y; M-81 y; M-83 y; F-87 y) and 4 *APOE* ϵ4,4 (M-70 y; M-78 y; F-79 y; M-85 y)]. As with the processing of the tissue samples from our epilepsy patients, autopsy tissue samples collected less than eight-hours postmortem from Alzheimer and control patients were identical, i.e., analogous samples to be used for molecular analyses were snap frozen in liquid nitrogen and for immunohistochemical analyses sections of formalin fixed brains were used.

Surgical waste and autopsy tissue are both exempt from IRB review under 46.101 5(b), and this study was approved as an exempt study by the University of Arkansas Institutional Review Board.

For further analysis of data obtained from epilepsy patients, we categorized genotypes into four groups. Group 1: patients having one or two alleles of ϵ2 and no ϵ4 [*APOE* ϵ2,2 (n = 1) and *APOE* ϵ2,3 (n = 12)]; Group 2: [*APOE* ϵ3,3 patients (n = 53)]; Group 3: patients having one ϵ4 allele [*APOE* ϵ2,4 (n = 2) *APOE* ϵ3,4 (n = 17)]; Group 4: [*APOE* ϵ4,4 patients (n = 7)]. This grouping allowed for investigation of the degree of influence of each of the three *APOE* alleles on observed results.

### Immunofluorescence immunohistochemistry

Reagents: Antibodies: rabbit anti-human IL-1α (Peprotech 4:1000): goat anti-human ApoE (Invitrogen 1:50): mouse anti-human Aβ/βAPP (Covance 1:1000); rabbit anti-human Pan Neuronal Marker (PNM – Millipore ABN 1:500); mouse anti-human DNA/RNA oxidative damage antibody (8-OH-gaunosine – Stress Marq Biosciences 5 μg/ml); rabbit anti-phosphorylated tau (AT8 – Abcam 1:3000); rabbit anti-actin (Santa Cruz 1:1000) were diluted in antibody diluent (DAKO). Secondary antibodies were Alexa Fluor® 488: donkey anti-rabbit or goat anti-rabbit; Alexa Fluor® 594: donkey anti-goat or goat anti-mouse. Mounting media containing Prolong Gold anti-fade reagent with DAPI (Invitrogen) was used to stain nuclei.

### Procedures

Paraffin-embedded tissue was sectioned at 7 μm and processed as previously described [2]. Sections destined for IL-1α, PNM, Stress Marq, and Aβ immunoreaction were pretreated by placing them in boiling sodium citrate buffer (0.01 M, pH 6.0) for 20 minutes; sections for ApoE immunoreaction were placed in trypsin solution for 10 min at 37°C, and all were blocked using protein block (DAKO), and immunoreacted by overnight incubation at room temperature. Appropriate Alexa Fluor-tagged secondary antibodies were diluted in antibody diluent 1:200, and sections were incubated for 60 minutes, washed three times for 5 minutes each in distilled water, and coverslipped with prolong Gold with DAPI.

### Image analysis

As previously described [2,15], a quantitative approach was used to assess numbers of glia and neurons. Three images per slide (40× magnification) were captured at identical exposure settings using a Nikon Eclipse E600 microscope equipped with a Coolsnap monochrome camera. Each of the three images, spanning 37,638.6 μm^2^, was acquired, analyzed, and thresholded using NIS-Elements BR3 software (Nikon.com). Results regarding neuronal and glial numbers are presented as numbers/mm^2^. Data were analyzed by ANOVA to assess differences among groups. Significance was provided by p ≤ 0.05.

### Real time (RT) polymerase chain reaction (PCR) amplification

Total RNA was extracted from brain tissue using TriReagent™ RNA (Molecular Research Center, Cincinnati, OH), according to the manufacturer's instructions. Real-time RT-PCR was performed as previously described [15]. Briefly, for comparisons of mRNA levels among different RNA samples, RT reactions were performed simultaneously using reagents from Life Technologies (Grand Island, NY). RT-PCR was performed using reagents from SyberGreen Master Mix from Life Technologies (Grand Island, NY). The sequences of primers for ApoE, βAPP, α-secretase (ADAM 10), BACE 1, BACE 2, IL-1α, IL-1β, and S100B are given in Table 1. Equal amounts of RT-PCR from each sample were pooled to use for standard curve reaction with each primer set to verify linearity and a suitable slope. All given mRNA tissue levels are relative to 18s.

**Table 1:** Human gene sequences for ApoE, βAPP, α-secretase, BACE 1, BACE 2, IL-1α, IL-1β, and S100B, with PCR annealing temperatures and number of amplification cycles

**Gene analyzed**	**Human sequences**	**Annealing temp. (Co)**	**Cycle no.**
ApoE	F: CCCAGGTCACCCAGGAACT	60	40
	R: AGTTCCGATTTGTAGGCCTTCA		
βAPP	F: AACCACCGTGGAGCTCCTT	60	40
	R: ATGCCACGGCTGGAGATC		
α-secretase (ADAM 10)	F: AGTGTACGTGTGCCAGTTC	60	40
	R: TTGCAGGGTGATGGTTCG		
BACE 1	F: CAGTCAAATCCATCAAGGCAG	60	40
	R: GTTGGTAACCTCACCCATTAGG		
BACE 2	F: AGAGTATAACGCAGACAAGGC	60	40
	R: CCAGTCCAGAAACCATCAGAG		
IL-1 α	F: GGAGAGCATGGTGGTAGTAGCAA	60	40
	R: TGGCTTAAACTCAACCGTCTCTT		
IL-1 β	F: GCACGATGCACCTGTACGAT	60	40
	R: CACCAAGCTTTTTTGCTGTGAGT		
S100B	F: CCCCAGGGACTCTTGTTAACAG	60	40
	R: CACGGTGCACGCTTTATCAC		

### Western immunoblot assay

Proteins were extracted from brain tissue in a lysis buffer comprised of 20 mM Tris-HCl (pH 7.5), 150 mM NaCl, 1% Nonidet P40, 1 mM EGTA, 1 mM EDTA, and 1% sodium deoxycholate; lysate protein was quantified using a Micro BCA assay reagent kit (Pierce, Rockford IL) as described previously [2]. Aliquots (30 μg each for ApoE and 50 μg for caspase 3) were loaded onto a 4-12% Criterion^XT^ precast Gel Bis-Tris from Bio-Rad (Hercules, CA), subjected to electrophoresis at 80V for 3 h, and transferred to PVDF 0.45 μm Immobilon-FL (Millipore). Blots were blocked in I-Block Buffer (Applied Biosystem Inc., Bedford, MA) for 60 minutes, then incubated overnight at 4°C with either mouse monoclonal anti-ApoE (1:500) (Santa Cruz sc-58242) primary antibody, rabbit anti-human Caspase3\Cleaved Caspase 3 (1:500) (Cell Signaling # 9661-2), or rabbit anti-actin (Santa Cruz 1:1000), and incubated for 1 h at room temperature with alkaline phosphatase-conjugated secondary antibody (1:1000). For protein detection, we used ProteinSimple Multifluor Western Blotting Kit (Santa Clara, CA), and for image capture we used CellBioscience FluorChem Q digital imager (Santa Clara, CA). Autoradiographs were digitized and analyzed using NIH Image software, version 1.60.

### Statistical analysis

Data were analyzed using an unpaired *t*-test and Wilcoxon distribution score, and results are expressed as mean + SD. In the Wilcoxon distribution plot, the length of the box represents the interquartile range (the distance between the 25th and 75th percentiles), the symbol in the box interior represents the group mean, the horizontal line in the box interior represents the group median, the vertical lines (the *whiskers*) issuing from the box extend to the group minimum and maximum values. Values were considered significantly different when the p-value was ≤ 0.05.

## Abbreviations

Aβ: Amyloid β; AD: Alzheimer’s disease; ApoE: Apolipoprotein E; AU: Arbitrary units; βAPP: Amyloid β precursor protein; Stress Marq: 8-OH guanosine; IL-1: Interleukin-1.

## Competing interests

The authors declare that they have no competing interests.

## Authors’ contributions

OA conducted and helped design all experiments and analyses and interpretation of the data, and writing the manuscript. REM conducted neuropathological evaluations and contributed to interpretation of results and writing of the manuscript. FAB was the neurosurgeon who provided the tissue and reviewed the manuscript. WSTG helped design the study with OA, verified and helped with interpretation of the data, and contributed to the writing of the manuscript. All authors read and approved the final manuscript.
